# Artificial intelligence outperforms standard blood-based scores in identifying liver fibrosis patients in primary care

**DOI:** 10.1038/s41598-022-06998-8

**Published:** 2022-02-21

**Authors:** Victoria Blanes-Vidal, Katrine P. Lindvig, Maja Thiele, Esmaeil S. Nadimi, Aleksander Krag

**Affiliations:** 1 Applied AI and Data Science, The Mærsk Mc-Kinney Møller Institute, University of Southern Denmark, Odense, Denmark; 2 Danish Centre for Clinical Artificial Intelligence (CAI-X), University of Southern Denmark and Odense University Hospital, Odense, Denmark; 3 Department of Gastroenterology and Hepatology, Odense University Hospital, Odense, Denmark; 4 Department of Clinical Research, University of Southern Denmark, Odense, Denmark

**Keywords:** Hepatology, Machine learning

## Abstract

For years, hepatologists have been seeking non-invasive methods able to detect significant liver fibrosis. However, no previous algorithm using routine blood markers has proven to be clinically appropriate in primary care. We present a novel approach based on artificial intelligence, able to predict significant liver fibrosis in low-prevalence populations using routinely available patient data. We built six ensemble learning models (LiverAID) with different complexities using a prospective screening cohort of 3352 asymptomatic subjects. 463 patients were at a significant risk that justified performing a liver biopsy. Using an unseen hold-out dataset, we conducted a head-to-head comparison with conventional methods: standard blood-based indices (FIB-4, Forns and APRI) and transient elastography (TE). LiverAID models appropriately identified patients with significant liver stiffness (> 8 kPa) (AUC of 0.86, 0.89, 0.91, 0.92, 0.92 and 0.94, and NPV ≥ 0.98), and had a significantly superior discriminative ability (*p* < 0.01) than conventional blood-based indices (AUC = 0.60–0.76). Compared to TE, LiverAID models showed a good ability to rule out significant biopsy-assessed fibrosis stages. Given the ready availability of the required data and the relatively high performance, our artificial intelligence-based models are valuable screening tools that could be used clinically for early identification of patients with asymptomatic chronic liver diseases in primary care.

## Introduction

The prevalence of fatty liver disease is increasing at the same rate as the epidemics of obesity and type 2 diabetes mellitus^1^. As the condition develops, inflammation and fibrosis of the liver appear and can progress to cirrhosis with associated morbidity and mortality. Early identification of asymptomatic patients can prevent that undetected liver fibrosis slowly and asymptomatically progresses to a severe and life-threatening chronic liver disease. Various tools for the non-invasive assessment of fibrosis have been developed in the last decades. They fall into three categories, 1) image-based technologies e.g. Transient Elastography (TE), that measures liver stiffness, 2) indirect markers e.g. Fibrosis 4 index (FIB-4), and 3) direct markers of fibrosis e.g. Enhanced Liver Fibrosis test (ELF). One of the main advantages of the indirect blood-based biomarkers and scores is that they can be used in evaluating liver fibrosis in most clinical settings, since they are very easy-to-use, quick and inexpensive. While these tools were originally developed, largely fueled by the hepatitis-C era, to diagnose significant fibrosis up-to compensated advanced chronic liver disease, they have primarily been validated in secondary and tertiary healthcare. Identifying those with significant liver fibrosis in primary care is a clinical challenge as the vast majority are asymptomatic and often have normal liver function tests. To date no tools have been developed and only few assessed or validated for a primary care population.

Up to now, algorithms based on indirect blood markers, have been typically developed using approaches that are standard in the epidemiological literature, such as parametric regression methods where the optimal set of predictors is identified by stepwise selection^2–5^. With the advent of computer systems and digitalization, large amount of healthcare data is routinely being collected and stored; but these data are frequently underused and undervalued. Artificial intelligence (AI) could help to improve the accuracy with which we can detect liver fibrosis by processing and automatically learning associations in this complex healthcare data. Among the different AI techniques, the use of ensemble learning is of particular interest, because it trains multiple machine learning algorithms, determines the optimal weights for combining the predictions, and results in an ensemble model that often outperforms any single machine learning algorithm. Despite the potential benefits, no studies have explored the use of ensemble learning methodology to identify significant liver fibrosis.

In this study, we developed a set of AI algorithms with different complexities, based on ensemble learning methodology, able to predict clinically significant liver stiffness (a well-established surrogate of biopsy-assessed liver fibrosis), using patient data that can be available at a low cost in primary care. We assessed their diagnostic performance, and conducted a head-to-head comparison with standard indirect indices (FIB-4, Forns index (Forns) and AST to platelet ratio index (APRI)). We then evaluated the ability of the ensemble learning models to effectively reduce the number of patients that undergo unnecessary TE investigations. Finally, we addressed the question as to whether a negative ensemble learning model result, calculated based on affordable patient data, could be used with confidence to rule out significant biopsy-assessed fibrosis, and so its potential role in eliminating unnecessary liver biopsies.

## Methods

### Study population

We performed a prospective cohort study including patients from the Region of Southern Denmark between 2013 and 2020. The study population (n = 3460) consisted of subjects at risk of NAFLD (~ 43% of the participants), subjects at risk of alcohol-related liver disease (ALD) (~ 35%), and subjects randomly selected from the general population (~ 22%). We recruited subjects via three main channels: 1) invitation letters sent to randomly selected Danes, from the Odense University Hospital’s catchment area by means of e-boks. E-boks is the official digital communication route between public authorities and Danish citizens; 2) from three alcohol rehabilitation centers; and 3) from in- and out-patients at the department of Gastroenterology and Hepatology at the Odense University Hospital of Southern Denmark. The non-participation rate was approximately 77% among subjects approached using channel 1 (i.e. general population), and 35% among subjects approached via channel 2 and 3.

At recruitment, none of the subjects had known liver diseases, and they were asymptomatic. We have published detailed study methods elsewhere^6,7^. All methods were carried out in accordance with relevant ethical guidelines and regulations based on the Declaration of Helsinki. We obtained informed consent in writing from each patient. The ethics committee in the Region of Southern Denmark (S-20120071; S-20170087) approved the study.

### Input and output variables

We collected data on demographics, physical exam, clinical and laboratory parameters, and questionnaires, alongside comorbidities and medications, resulting in 233 potential input variables (Appendix). Liver stiffness measurement (LSM) assessed by TE using FibroScan, was dichotomized using an 8 kPa threshold according to the definition of clinically significant fibrosis^8,9^.

The objective was to build a model that, when applied to undiagnosed patients that are new to the model (unseen data), correctly classifies them into two classes: patients whose liver stiffness is expected to be > 8 kPa (i.e. “clinically significant LSM”) and patients with expected liver stiffness ≤ 8 kPa (i.e. “not clinically significant LSM”).

**Figure 1:**
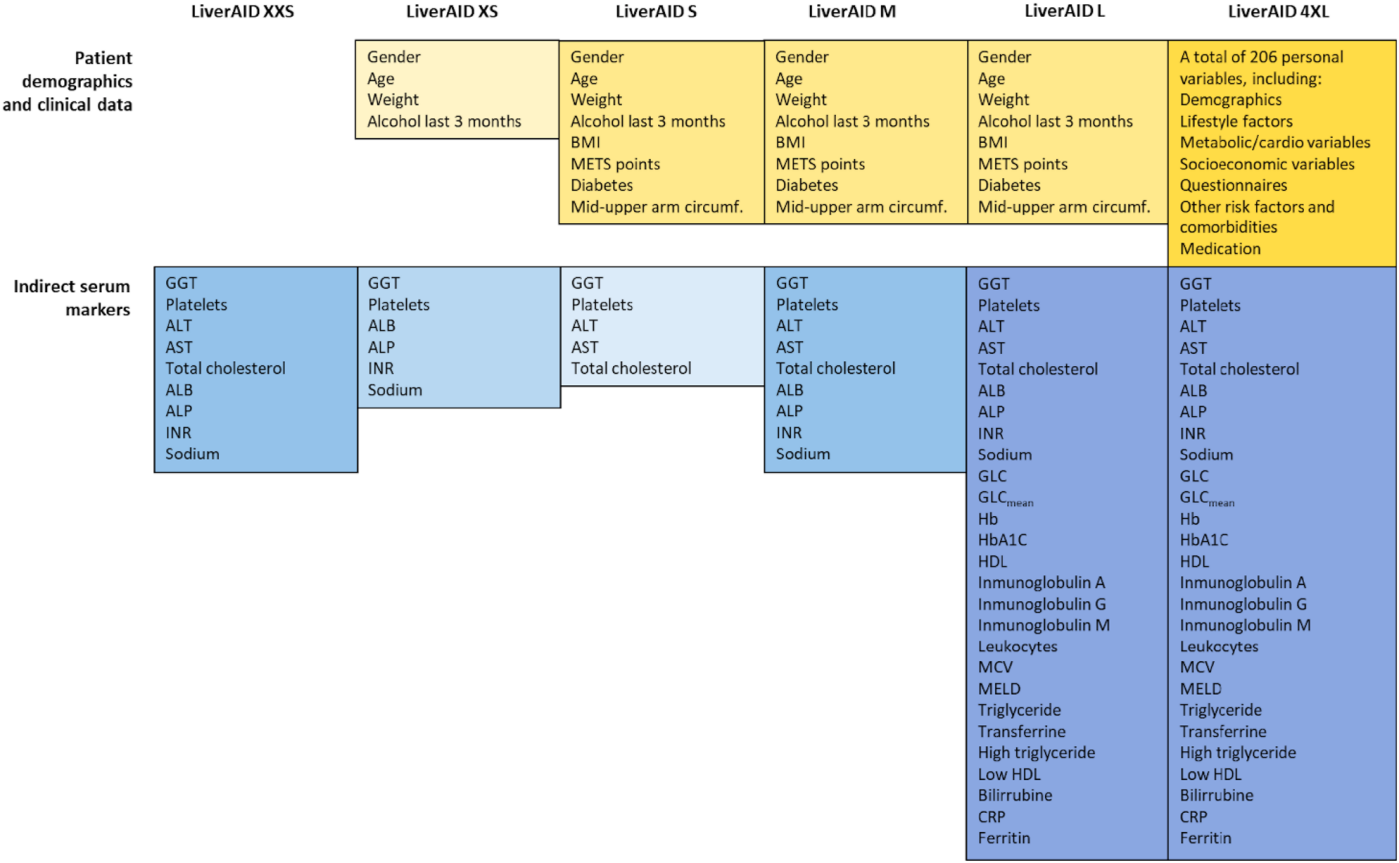
Patients demographic information, clinical data and indirect serum markers used as input parameters in each of the LiverAID models.

Our model should ideally strike a balance between model complexity and performance. Therefore, we built six different ensemble learning models (LiverAID models), of increasing complexity, in terms of the number of input variables required by the model (Fig. 1). LiverAID XXS relies exclusively on the 9 indirect blood-based biomarkers used in the calculation of standard indices (FIB-4, Forns, APRI and LiverTrail)^2–4,6^. (Fig. 1). LiverAID XS uses as inputs the 4 clinical variables and 6 indirect blood-based markers proposed in Thiele et al.^6^. Models LiverAID S, M and L, use as inputs the same 8 clinical variables (i.e. gender, age, weight, alcohol consumption in the last 3 months, BMI, metabolic syndrome (METS) points, diabetes and mid-upper arm circumference), but they differ in which blood biomarkers are additionally included as inputs: LiverAID S includes the 5 serum markers used in FIB-4, Forns and APRI indexes^2–4^; LiverAID M includes the 9 serum markers used in FIB-4, Forns, APRI and LiverTrail^2–4,6^; while LiverAID L includes 27 routine blood markers (Fig. 1). Finally, LiverAID 4XL, uses as inputs all 233 available variables, i.e. the same 27 routine blood markers used in LiverAID L, plus a comprehensive set of 206 demographic and clinical variables (Appendix).

### The ensemble learning models

We generated high-performance classifiers following an ensemble learning strategy^10^. Based on De Lell et al.^11^, each of our proposed ensemble learning models were built to combine and optimize the output from four basic learning algorithms (random forest, elastic net, bagging classification tress and support vector machine) (Appendix).

**Figure 2:**
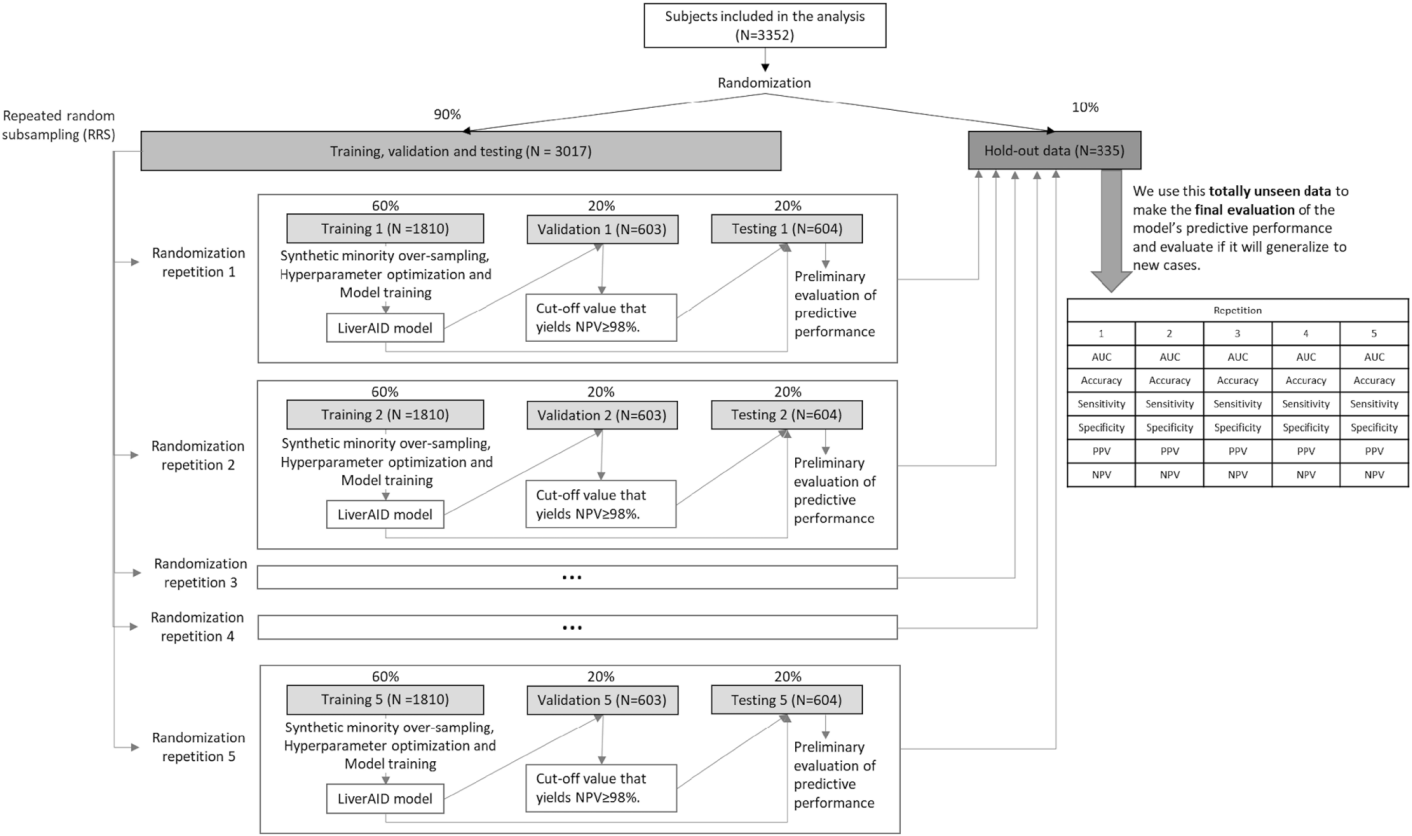
Data splitting including repeated random subsampling approach and final evaluation in the hold-out dataset.

With a view to reliably assessing the generalization error, we performed a series of data splits (Fig. 2). We randomly extracted a subset (10%) of the data (hold-out dataset), which was not used in any way for training, validation or testing. This unseen data was exclusively reserved to perform the final assessment of the model’s predictive performance. We applied a repeated random subsampling (RRS) strategy in which the remaining dataset was repeatedly and randomly split (resulting in 5 repetitions) into training (60%), validation (20%) and testing (20%). Synthetic samples were generated, only in the training dataset, to compensate for class imbalance. The optimal values for the hyperparameters of each model were obtained using the training dataset (Fig. 2). The validation datasets were then used to optimize the probability threshold value that resulted in an NPV ≥ 98%, while the testing datasets were used for a preliminary estimation of the generalization error. Finally, the predictive performance of each model was evaluated on the completely unseen hold-out dataset.

The ability of standard indices of liver fibrosis (APRI, FIB-4 or Forns index) to predict significant liver stiffness (i.e. LSM ≤ 8 kPa vs. LSM > 8 kPa) was evaluated by four logistic regressions models built using the same training datasets as in the LiverAID models: Three univariate models, using respectively, APRI, FIB-4 or Forns index as predictors; and one multivariate model with all three indices as predictors. We used the hold-out dataset to perform a head-to-head comparison of the predictive performance of each LiverAID model and that of the logistic regression models. We conducted all analyses in R.

### Liver biopsy

A portion of the study population was at a significant risk of liver disease that justified performing a liver biopsy^6,7^. We performed a percutaneous liver biopsy to these patients^6,7^, and obtained the liver fibrosis Kleiner stage (F0 to F4). We further evaluated the ability of: 1) LSM; 2) APRI, FIB-4 and Forns approaches, and 3) LiverAID models; to rule out significant liver fibrosis (defined as F2-F4 kleiner stages) in the hold-out dataset, using biopsy-assessed fibrosis stage as the reference standard. In this evaluation we mapped liver stiffness ≤ 8 kPa, to liver biopsy stage F0 and F1; and liver stiffness > 8 kPa to liver biopsy stage F2 to F4, where liver stiffness was measured in case (1) and predicted in cases (2) and (3).

### Ethics committee approval

The ethics committee in the Region of Southern Denmark (S-20120071; S-20170087) approved the study.

## Results

### Study population

Subject demographics, LSM results, biopsy-assessed fibrosis stage and serum markers measurements are presented in Table 1.

**Table 1:** – Subjects characteristics.

Characteristic	Units or levels	Summary data (n = 3460)
**Liver stiffness**		
LSM^1^	kPa	4.6 ± 2.1
Liver stiffness status based on LSM^2^	significant/not significant	403/2949
**Demographics**		
Sex	male/female	1584/1768
Age	years	57 ± 13
Weight	kg	83 ± 25
Alcohol consumption in the last 3 months	units/week	6 ± 15
BMI		27.3 ± 7
METS points	0/1/2/3/4/5	336/753/824/608/448/383
Diabetes	no/yes	3023/329
Mid-upper arm circumference	cm	30 ± 5
**Serum markers**		
ALT	U/L	26 ± 16
AST	U/L	25 ± 11
ALP	g/L	69 ± 28
GGT	U/L	29 ± 36
ALB	g/L	45 ± 4
INR		1.0 ± 0.1
Bilirubine	µmol/L	8 ± 6
Platelets	10^9/L	242 ± 75
Hb	mmol/L	8.8 ± 1.0
MCV	fL	90 ± 5
Leukocytes	10^9/L	6.0 ± 2.3
CRP	mg/L	1.5 ± 2.7
Ferritin	µg/L	145 ± 173
Sodium	mmol/L	140 ± 3
Cholesterol	mmol/L	5.0 ± 1.4
Triglycerides	mmol/L	1.1 ± 0.8
Elevated triglycerides	no/yes	2208/1144
HDL	mmol/L	1.5 ± 0.6
Low HDL cholesterol	no/yes	2397/955
HbA1c	mmol/mol	36 ± 6
GLC	mmol/L	5.7 ± 0.8
GLC_mean_	mmol/L	6.1 ± 0.8
IgA	g/L	2.2 ± 1.4
IgG	g/L	10.1 ± 2.9
IgM	g/L	0.86 ± 0.64
MELD		6 ± 1
**Biopsy**	**Units or levels**	**Summary data** (N = 463)
Biopsy-assessed fibrosis stage	F0/F1/F2/F3/F4	41/168/139/50/65

All summary data are medians ± interquartile range or counts (%).

^1^LSM = Liver stiffness measurement (median of 10 valid transient elastography measurements).

^2^Number of subjects whose LSM is > 8 kPa (corresponding to “significant liver stiffness”) and subjects whose LSM is ≤ 8 kPa (corresponding to “not significant liver stiffness”).

ALB, Albumin; ALP, Alkaline phosphatase; GGT, Gamma-Glutamyltransferase; INR, International Normalised Ratio; ALT, Alanine aminotransferase; AST, Aspartate aminotransferase; CRP, C-reactive protein; GLC, Fasting glucose; GLC_mean_, Mean glucose calculated from HbA1C; Hb, Hemoglobine; HbA1c, Hemoglobin A1c; HDL, HDL cholesterol; MCV, Mean corpuscular volume; MELD, Model of End-stage Liver Disease.

A total of 3352 patients had a valid liver stiffness measurement and 463 patients also underwent a liver biopsy investigation. All demographic and serum variables included in the analysis had ≤ 15% missing values. Missing values in these variables were handled by creating imputations (replacement values) for these multivariate missing data.

### Area Under the Curve (AUC) of the Receiver Operating Characteristic curve (ROC) of LiverAID models predicting significant liver stiffness (LSM > 8 kPa) in training, validation, testing and hold-out datasets

The AUC in the training set was, for all models, equal to 1.00. When tested on the validation, testing and hold-out datasets, the LiverAID models subsequently achieved an AUC of 0.86, 0.87 and 0.86 (XXS), 0.88, 0.88 and 0.89 (XS), 0.89, 0.90 and 0.91 (S), 0.91, 0.91 and 0.92 (M), 0.91, 0.90 and 0.92 (L) and 0.93, 0.93 and 0.94 (4XL), respectively (Appendix). For each model (XXS to 4XL), the discriminatory power of the models built from each of the different training subset data was comparable, with minimal variability in AUC values between repetitions (Appendix).

### Head-to-head comparison of LiverAID models vs standard blood-based indices in predicting significant liver stiffness (LSM > 8 kPa)

The accuracy, sensitivity, specificity, PPV and NPV of using: 1) a cut-off approach for standard blood-based indices, 2) logistic regression models with standard blood-indices as predictors, and 3) ensemble learning models, is shown in Table 2. In the first approach, low cut-off values (i.e. used to rule out the presence of significant fibrosis) of FIB-4 = 1.25^3^, Forns = 4.1^2^ and APRI = 0.5 ^4^, were used, which resulted in 9, 27 and 10 missed diagnosis (patients with significant LSM) for every 100 patients with FIB-4 < 1.25, Forns < 4.1 or APRI < 0.5, respectively. In the second and third approach, probability thresholds for each model were optimized in the validation dataset and then directly applied to the hold-out data set to evaluate model’s performance. Our goal was, in all logistic and LiverAID models, to determine probability cut-off values that result in NPV ≥ 0.98 in the validation dataset (i.e. ≤ 2 patients with missed diagnoses for every 100 patients with a negative test result). However, logistic regression models could not achieve this targeted NPV, and in these cases, thresholds were chosen to obtain the maximum possible NPV. This resulted in a NPV of logistic models in the hold-out dataset of 0.91, 0.88, 0.91 and 0.92, and PPV of 0.31, 0.57, 0.36 and 0.33, when using as predictors FIB-4, Forns, APRI, and FIB4 + Forns + APRI, respectively (Table 2). On the contrary, in the case of LiverAID models, it was possible to achieve NPV ≥ 0.98 in the validation dataset. In the hold-out dataset, the NPV of all LiverAID models remained ≥ 0.98, and the PPV was 0.22, 0.24, 0.30, 0.31, 0.31 and 0.35 (for LiverAID XXS, XS, S, M, L and 4XL, respectively) (Table 2). Diagnostic performance measures for the prediction of significant liver stiffness (LSM > 8 kPa) in the hold-out data set, for each subpopulation (i.e. subjects at risk of NAFLD, subjects at risk of alcohol-related liver disease, and subjects randomly selected from the general population) is shown in Table 3.

**Table 2:** - Diagnostic performance measures evaluated using the hold-out (completely unseen) dataset.

Type of method/model	Prediction of significant liver stiffness defined as measured liver stiffness (LSM) > 8 kPa (N = 335)							Prediction of significant liver fibrosis defined as Kleiner biopsy stage (F2 to F4) (N = 55)				
	Method/model	AUC	Accuracy	Sensitivity	Specificity	PPV	NPV	Accuracy	Sensitivity	Specificity	PPV	NPV
1. Cut-off values for the standard blood-based indices^1^	FIB-4	–	0.6	0.71	0.58	0.25	0.91	0.55	0.11	1.00	1.00	0.52
	Forns	–	0.17	0.93	0.03	0.15	0.73	0.71	0.82	0.59	0.68	0.76
	APRI	–	0.85	0.49	0.91	0.52	0.9	0.62	0.92	0.31	0.57	0.80
2. Logistic regression using standard blood indices as predictors	FIB-4	0.7	0.71	0.64	0.73	0.31	0.91	0.63	0.27	1.00	1.00	0.58
	Forns	0.6	0.86	0.27	0.96	0.57	0.88	0.69	0.57	0.81	0.76	0.65
	APRI	0.74	0.77	0.6	0.8	0.36	0.91	0.69	0.75	0.63	0.68	0.71
	FIB-4 + Forns + APRI	0.76	0.74	0.66	0.76	0.33	0.92	0.63	0.87	0.40	0.59	0.75
3. Ensemble learning models	LiverAID XXS	0.86	0.52	0.95	0.44	0.22	0.98	0.65	0.96	0.33	0.60	0.90
	LiverAID XS	0.89	0.56	0.95	0.49	0.24	0.98	0.56	0.96	0.16	0.54	0.79
	LiverAID S	0.91	0.68	0.92	0.64	0.3	0.98	0.61	0.96	0.26	0.57	0.85
	LiverAID M	0.92	0.69	0.9	0.66	0.31	0.98	0.61	0.96	0.26	0.57	0.85
	LiverAID L	0.92	0.69	0.92	0.65	0.31	0.98	0.61	0.98	0.22	0.57	0.88
	LiverAID 4XL	0.94	0.74	0.94	0.71	0.35	0.99	0.62	0.97	0.25	0.57	0.89
4. Transient elastography	LSM	–	–	–	–	–	–	0.84	0.93	0.74	0.79	0.91

^1^Cut-off values of FIB-4 = 1.25, Forns = 4.1 and APRI = 0.5 were used.

**Table 3:** - Diagnostic performance measures for the prediction of significant liver stiffness defined as measured liver stiffness (LSM) > 8 kPa, evaluated using the hold-out (completely unseen) dataset, for each subpopulation: subjects at risk of NAFLD, subjects at risk of alcohol-related liver disease (ALD), and subjects randomly selected from the general population.

	Method/model	Accuracy	Sensitivity	Specificity	PPV	NPV
**NAFLD (N = 136)**						
1. Cut-off values for the standard blood-based indices^1^	FIB-4	0.82	0.00	1.00	–	0.82
	Forns	0.22	0.91	0.06	0.18	0.75
	APRI	0.86	0.48	0.95	0.67	0.89
2. Logistic regression using standard blood indices as predictors	FIB-4	0.74	0.52	0.78	0.35	0.88
	Forns	0.83	0.23	0.97	0.63	0.85
	APRI	0.76	0.48	0.83	0.38	0.88
	FIB-4 + Forns + APRI	0.76	0.64	0.78	0.39	0.91
3. Ensemble learning models	LiverAID XXS	0.61	0.96	0.54	0.30	0.98
	LiverAID XS	0.53	0.93	0.45	0.26	0.97
	LiverAID S	0.63	0.94	0.56	0.31	0.98
	LiverAID M	0.63	0.94	0.56	0.31	0.98
	LiverAID L	0.63	0.93	0.57	0.31	0.98
	LiverAID 4XL	0.68	0.92	0.62	0.34	0.98
**ALD (N = 114)**						
1. Cut-off values for the standard blood-based indices^1^	FIB-4	0.82	0.15	1.00	1.00	0.81
	Forns	0.18	0.94	0.01	0.18	0.50
	APRI	0.79	0.55	0.85	0.50	0.88
2. Logistic regression using standard blood indices as predictors	FIB-4	0.67	0.75	0.65	0.37	0.91
	Forns	0.84	0.39	0.94	0.58	0.87
	APRI	0.69	0.70	0.69	0.38	0.89
	FIB-4 + Forns + APRI	0.67	0.67	0.67	0.33	0.89
3. Ensemble learning models	LiverAID XXS	0.59	0.90	0.52	0.30	0.96
	LiverAID XS	0.51	0.99	0.40	0.27	0.99
	LiverAID S	0.64	0.90	0.58	0.33	0.96
	LiverAID M	0.64	0.90	0.58	0.33	0.96
	LiverAID L	0.64	0.93	0.57	0.33	0.98
	LiverAID 4XL	0.68	0.99	0.60	0.36	1.00
**General population (N = 85)**						
1. Cut-off values for the standard blood-based indices^1^	FIB-4	0.94	0.00	1.00	-	0.94
	Forns	0.07	1.00	0.01	0.05	1.00
	APRI	0.90	0.25	0.94	0.20	0.96
2. Logistic regression using standard blood indices as predictors	FIB-4	0.74	0.75	0.74	0.14	0.98
	Forns	0.93	0.00	0.99	0.00	0.95
	APRI	0.88	0.75	0.88	0.27	0.98
	FIB-4 + Forns + APRI	0.79	0.68	0.79	0.22	0.96
3. Ensemble learning models	LiverAID XXS	0.84	0.75	0.84	0.19	0.99
	LiverAID XS	0.68	0.90	0.67	0.12	0.99
	LiverAID S	0.87	0.75	0.88	0.23	0.99
	LiverAID M	0.87	0.75	0.88	0.23	0.99
	LiverAID L	0.86	0.75	0.87	0.22	0.99
	LiverAID 4XL	0.95	0.75	0.96	0.47	0.99

The AUC in the hold-out dataset, of univariate logistic models using standard indices as predictors was 0.70 (95%CI: 0.60–0.80, p < 0.001) (for FIB-4), 0.60 (95%CI: 0.49–0.71, *p* < 0.001) (for Forns) and 0.74 (95%CI: 0.65–0.83, *p* < 0.001) (for APRI), showing no variability among repetitions. The AUC of multivariate models using all three indices as predictors, ranged from 0.75 (0.65–0.84) (repetition 2) to 0.77 (0.67–0.86) (repetition 3), while the independent association between indices and significant LSM was p_FIB-4_ < 0.001, p_Forns_ < 0.05 and p_APRI_ < 0.001.

**Figure 3:**
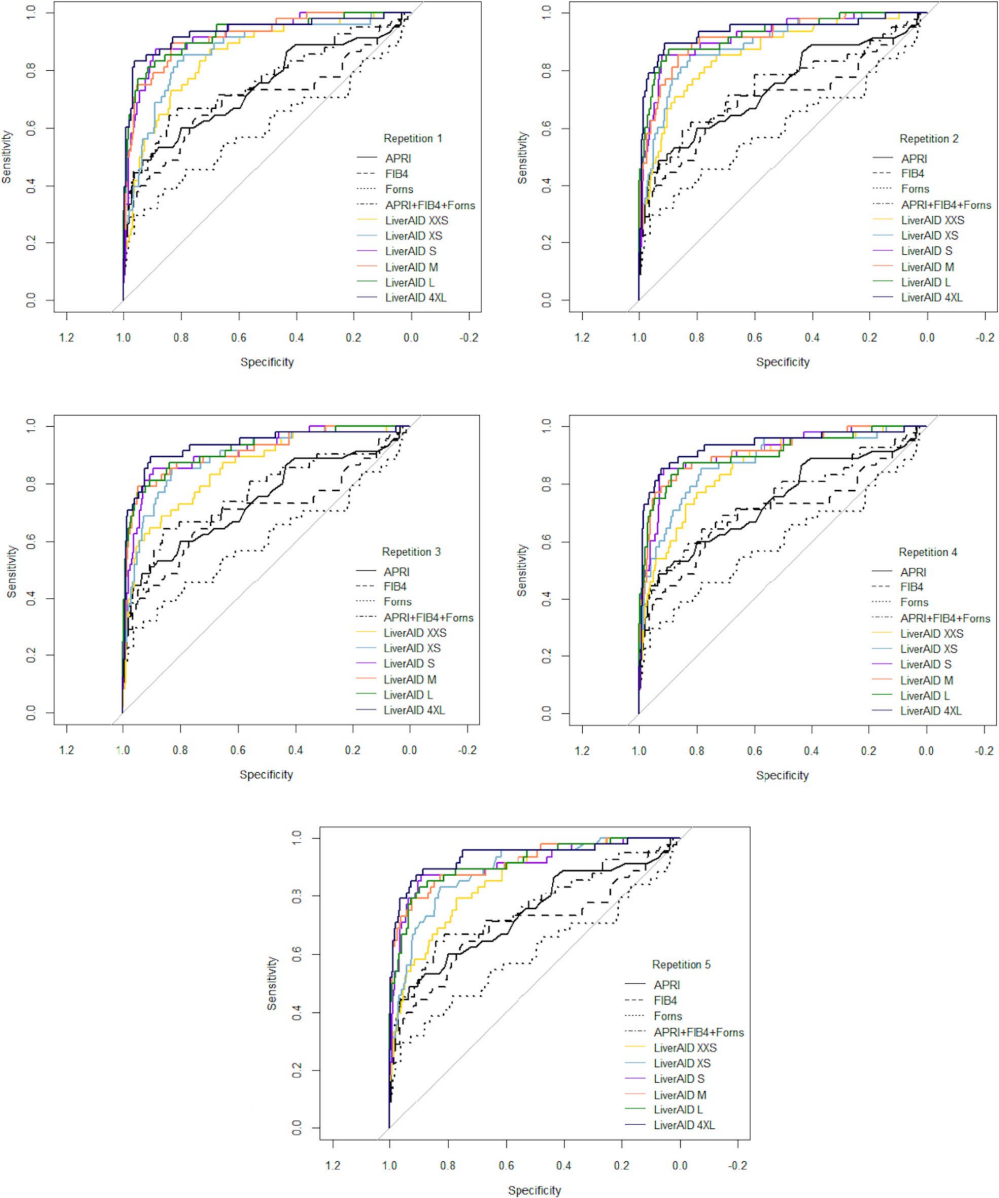
Receiver operating characteristic (ROC) curves for the prediction of clinically significant liver stiffness (> 8 kPa) in the hold-out dataset, repetition 1 to 5 respectively (n = 335) of LiverAID models (LiverAID XXS, XS, S, M, L, 4XL) and logistic regressions using standard blood-based indices of liver fibrosis as predictors (univariate FIB-4, Forns, APRI and multivariate FIB-4 + Forns + APRI).

The diagnostic performance for the detection of significant liver stiffness (> 8 kPa) in the hold-out data set, of all LiverAID models (AUC_LiverAID_XXS_ = 0.86, AUC_LiverAID_XS_ = 0.89, AUC_LiverAID_S_ = 0.91, AUC_LiverAID_M_ = 0.92, AUC_LiverAID_L_ = 0.92, AUC_LiverAID_4XL_ = 0.94) was in all cases and repetitions significantly higher than that of any regression model based on standard blood-based indices (AUC_FIB-4_ = 0.70, AUC_Forns_ = 0.60, AUC_APRI_ = 0.74 and AUC_FIB-4+Frons+APRI_ = 0.76) (p < 0.01) (Fig. 3 and Table 4). When comparing LiverAID models among themselves, the difference in classification performance between LiverAID XXS and XS was not statistically significantly (p > 0.10). Similarly, LiverAID S, M and L did not significantly differ from each other (p > 0.10). The classification performance of LiverAID S, M and L was, in the great majority of the cases/repetitions (93%), significantly better than LiverAID XXS (p < 0.05), and in most cases/repetitions, significantly better than LiverAID XS (p < 0.05), but this last result did not stay consistent throughout (Table 4 and Appendix). Finally, model LiverAID 4XL clearly outperformed XXS and XS (p < 0.05), but it showed contrasting results when compared to LiverAID S, M and L (p = 0.014–0.219) (Table 4 and Appendix).

**Table 4:** - P-values for the comparison of AUC between LiverAID models and standard blood-based indices in predicting significant liver stiffness (LSM > 8 kPa).

	FIB-4	Forns	APRI	FIB-4 + Forns + APRI	LiverAIDXXS	LiverAIDXS	LiverAIDS	LiverAIDM	LiverAIDL	LiverAID 4XL
AUC	0.70	0.60	0.74	0.76	0.86	0.89	0.91	0.92	0.92	0.94
FIB-4	–									
Forns	0.987	–								
APRI	0.867	0.998	–							
FIB-4 + Forns + APRI	0.021	0.003	0.173	–						
LiverAID XXS	0.000	0.000	0.000–0.001	0.002–0.008	–					
LiverAID XS	0.000	0.000	0.000–0.001	0.001–0.008	0.065–0.430	–				
LiverAID S	0.000	0.000	0.000	0.000–0.001	0.005–0.031	0.000–0.102	–			
LiverAID M	0.000	0.000	0.000	0.000	0.001–0.016	0.003–0.132	0.300–0.485	–		
LiverAID L	0.000	0.000	0.000	0.000	0.004–0.060	0.001–0.067	0.187–0.482	0.172–0.440	–	
LiverAID 4XL	0.000	0.000	0.000	0.000	0.002–0.006	0.001–0.041	0.044–0.219	0.049–0.126	0.014–0.157	–

Ranges indicate the minimum and maximum *P*-values from the repeated models (repetitions 1 to 5). The results for each repetition can be found in Appendix.

### Head-to-head comparison of LiverAID models, transient elastography and standard blood-based indices in predicting significant biopsy-assessed liver fibrosis stage (F2-F4)

In the patients from the hold-out dataset that underwent a liver biopsy (N = 55), LSM (≤ 8 kPa vs. > 8 kPa) was able to predict biopsy fibrosis stage (F0-F1 vs. F2-F4) with a NPV = 0.91 (Table 3). Liver stiffness (≤ 8 kPa vs. > 8 kPa) estimated with LiverAID XXS, XS, S, M, L and 4XL was able to predict biopsy fibrosis stage (F0-F1 vs. F2-F4) with a NPV of 0.90, 0.79, 0.85, 0.85, 0.88 and 0.89, respectively (Table 3). In comparison, liver stiffness (≤ 8 kPa vs. > 8 kPa) estimated from standard blood-based indices was able to predict biopsy fibrosis stage with NPV of 0.52, 0.76 and 0.80 (for FIB-4, Forns and APRI cut-off approach) and NPV of 0.58, 0.65, 0.71 and 0.75 (for FIB-4, Forns, APRI and FIB-4 + Forns + APRI logistic regression approach) (Table 2).

## Discussion

Our study demonstrates that ensemble learning models that use routinely available clinical data as inputs, are able to appropriately detect clinically significant liver fibrosis in low-prevalence settings. In comparison to models using traditional regression techniques and standard blood-based indices, our strategy using ensemble learning demonstrated significantly better diagnostic performance.

Model selection techniques should find an optimal trade-off between the ability of the model to fit data and the model’s required complexity to do so. In relation to this, much emphasis has been placed in the literature on obtaining predictive models for liver fibrosis that are “simple”^12^. Strictly speaking, complexity can be separated into the model-complexity and the inputs-complexity dimension. Briefly, the model-complexity dimension pertains to how complex is the model itself, which affects, among others, the prediction time. In praxis, differences in prediction time are inconsequential, because with the current computational power of personal computers and the availability of cloud computing, the result on the predicted status of fibrosis for each new patient can be in all approaches considered in this article, available for the clinician at the click of a button. The inputs-complexity dimension pertains to how many input parameters on each new patient are required by the model to make a prediction, and how costly and feasible is obtaining information on these specific parameters in clinical settings. All our LiverAID models involve objective and readily available laboratory variables and non-invasive clinical information; and none of them require information from invasive or resource intensive procedures. In this regard, they are therefore more advantageous than other methods such as ELF or TE. The question remains now how do the LiverAID models compare to each other and to traditional blood-based indices. Whilst acknowledging the very high performance of LiverAID 4XL (AUC = 0.94), this comprehensive model will not be, most likely, the “method of choice”, since it requires for the clinician to obtain and entry into the model a large amount of non-automated information from each investigated patient, which is very time-consuming, and entails a high risk of introducing operator errors.

Placing greater value on simplicity and usability, the remaining models (XXS to L) may then be considered. Models LiverAID L and M do not imply a significantly better performance compared to LiverAID S; while LiverAID XS did not outperform LiverAID XXS. Therefore, two models stand out as being especially efficient in their ability to predict significant liver stiffness: LiverAID XXS (AUC = 0.86) and LiverAID S (AUC = 0.91). The main advantage of LiverAID XXS is that it exclusively relies on data from 9 objective laboratory markers obtained from routine blood tests. This could be particularly valuable when aiming at identifying people with asymptomatic liver disease in the general population. No demographic or clinical information about the patient is required, beyond these 9 serum markers; thereby releasing resources (i.e. the cost of performing history taking and physical examination) that could be used in increasing the population undergoing laboratory testing for initial screening for liver fibrosis. In comparison, LiverAID S relies on only 5 serum markers, but it requires some basic demographic and clinical information about the patient. It should be highlighted though, that LiverAID S performed significantly better than LiverAID XXS, while still affording a reasonable balance between the goodness of fit and number of parameters required by the model. As to FIB-4, Forns and APRI regression models, these require data on 2–6 parameters. However, the performance of these traditional approaches was also markedly inferior (AUC = 0.60–0.76, vs. AUC = 0.86–0.91, p = 0.000–0.001).

One of the most important aims of screening for liver fibrosis is to make clinical decisions about further diagnostic tests and possibly treatments, by excluding subjects with zero-to-little fibrosis. We address the question of how many subjects testing negative using each of the investigated methods, do have significant liver stiffness, despite having negative test results. Standard blood-based indices, commonly used in clinical practice, performed poorly in identifying candidates at low risk of having significant liver fibrosis in that: 9–27% of patients with lower scores than the proposed cut-off threshold, and 8–12% of patients with a negative logistic regression result based on FIB-4, Forns and/or APRI as predictors; had LSM > 8 kPa. In comparison, all LiverAID models could achieve a NPV ≥ 0.98. With a negative result overlooking the existence of significant LSM in only ≤ 2% of the patients, we could focus on what the test means in patients with positive test results. In a clinical context, a patient with a positive LiverAID test would be referred for further investigations (e.g. TE). A patient classified as positive using LiverAID XXS (i.e. predicted LSM > 8 kPa) has a 22% probability of subsequently obtaining an actual LSM > 8 kPa and a 78% probability of obtaining LSM ≤ 8 kPa (i.e. unnecessary TE examination or over-testing). In the case of using LiverAID S, these probabilities are 30% and 70%, respectively. Consequently, using LiverAID S instead of LiverAID XXS, translate into an estimated reduction of 8% in the number of patients undergoing unnecessary transient elastography tests. The decision on using LiverAID S vs. LiverAID XXS may have thus economic implications for the healthcare system. These are currently undetermined since performing cost–effectiveness analyses of alternative health assessment strategies is beyond the scope of this paper. Finally, we evaluated the performance across different clinically relevant subgroups, i.e. subjects at risk of NAFLD, subjects at risk of alcohol-related liver disease (ALD), and subjects randomly selected from the general population. LiverAID models showed an adequate performance in each of the three subgroups. In LiverAID XXS and LiverAID S, the NPV’s were 0.98, 0.96 and 0.99, for NAFLD, ALD and general population subgroups, respectively. In LiverAID XXS, the PPV’s were 0.30, 0.30 and 0.19, for NAFLD, ALD and general population subgroups, respectively; while in LiverAID S, the PPV’s were 0.31, 0.33 and 0.23.

It is well established that LSM can be used as a surrogate of liver fibrosis, and that TE is a sensitive tool to be used, as a triage test before biopsy, for identifying patients that could obviate biopsy. If the NPV of LiverAID models in predicting significant biopsy-assessed liver fibrosis was sufficiently high in comparison with the NPV of TE, then in clinical practice a negative LiverAID result (instead of a negative TE result), could be used to avoid the need for liver biopsy. In the primary care clinical setting, which is the relevant implementation context for the LiverAID models, a slightly lower NPV of LiverAID, compared to TE would also be clinically acceptable. This is because in primary care, a clinical decision must be made as of whether a patient should be referred for further examinations or not (unlike in specialized departments, where often a decision has to be made regarding the need of performing a liver biopsy). Our study showed that, in the subgroup of patients who underwent a liver biopsy, 9% of the patients with a negative TE result had significant biopsy-assessed liver fibrosis (F2-F4 kleiner stage). In comparison, 10% and 15% of the patients with a negative LiverAID XXS and S result, had a F2-F4 kleiner stage of liver fibrosis. Therefore, LiverAID XXS and S showed a relatively good ability to reliably predict the absence of significant biopsy-assessed fibrosis stage (F2-F4), in patients who were assessed for suspected liver fibrosis using liver biopsy. We must point out that, in our study, a liver biopsy was performed when a subject had a LSM > 8 kPa; therefore, the biopsy subgroup cannot be considered representative of a “low prevalence population”.

One main limitation of our study is that validation was performed internally, using a random sample of the population of study. Although this data was not used at any stage during the model building process, further studies are needed to evaluate whether the models maintain their diagnostic performance in external populations. In the general population, the prevalence of significant liver stiffness (> 8 kPa) has been reported as 2–7.5%^13,14^, while prevalences have been reported as being 34% among patients with type 2 diabetes and 18.3% among hazardous alcohol users^8^. The prevalence observed in our study (13%) accords with the fact that, in our cohort, the frequency of subjects with known risk factors for liver fibrosis is somewhat overrepresented compared to the general population. This is both deliberate and clinically justified, since, in clinical practice, targeting patients with known risk factors is a more effective screening strategy for identification of patients with asymptomatic chronic liver disease^8^. It is worth noting that in a diminished prevalence setting (i.e. general population), the true ability of a negative LiverAID test to rule out significant fibrosis will be increased, as well as the number of unnecessary TE examinations. For future research, it would be desirable to validate the LiverAID models in a low prevalence cohort with liver biopsy as the reference standard.

In conclusion, we present a set of AI-based models with different complexities, able to successfully predict clinically significant liver fibrosis, using patient data that can be available at a low cost in clinical settings. Two of our ensemble models stand out as being especially efficient, one requiring 9 routine serum markers, and another one requiring 5 routine serum markers and 8 basic demographic/clinical variables. Given the ready availability of the required data, along with the relatively high accuracy in separating patients’ risks, our ensemble models seem to be valuable and practical tools that could be used clinically for early identification of patients with asymptomatic chronic liver diseases in primary care.

## Supplementary Information

Supplementary Information.

## Data Availability

The source data for this article cannot be made freely open access as it is considered pseudonymised data. Access to data can be granted via contact with the corresponding author and after approval from the Danish Data Protection Agency.

## Abbreviations

TE: Transient elastography
FIB-4: Fibrosis 4 index
ELF: Enhanced liver fibrosis test
PPV: Positive predictive value
NPV: Negative predictive value
AI: Artificial intelligence
Forns: Forns index
APRI: AST to platelet ratio index
ALD: Alcohol-related liver disease
BMI: Body mass index
LSM: Liver Stiffness Measurement
KpA: Kilopascal
GGT: Gamma-glutamyl transferase
ALT: Alanine transaminase
AST: Aspartate transaminase
ALB: Albumin
ALK: Alkaline phosphatase
INR: International normalized ratio
METS: Metabolic syndrome
AUC: Area under the curve
RRS: Random subsampling
SMOTE: Synthetic minority over-sampling technique
ROC: Receiver operating characteristic curve
