# Supplementary material for: Artificial intelligence outperforms standard blood-based scores in identifying liver fibrosis patients in primary care
Source: Sci Rep. 2022 Feb 21;12:2914. doi: 10.1038/s41598-022-06998-8 (PMC8861108; doi:10.1038/s41598-022-06998-8)
Supplement: Supplementary file 1 — Supplementary Information. [file 41598_2022_6998_MOESM1_ESM.docx]

**APPENDIX**

**1. Complete list of 234 variables, including 1 outcome variable (Variable 1) and 233 potential input variables (Variables 2 to 234)**

| **Variable** | **Label** |
| --- | --- |
| [1] "te_out" | Liver fibrosis status based on LSM, i.e. LSM >8 kPa (corresponding to “significant liver stiffness”) vs. LSM ≤8 kPa (corresponding to “not significant liver stiffness”) |
| [2] "sex" | sex |
| [3] "age" | age in years |
| [4] "age_cci" | age in categories for the Charlson comorbidity index |
| [5] "risk1___1" | alcohol as a risk factor (based on overuse variable) |
| [6] "med_alc___1" | disulfiram |
| [7] "med_alc___2" | acamprosate |
| [8] "med_alc___3" | naltrexone |
| [9] "med_alc___999" | other alcohol treatment |
| [10] "smoke" | Do you smoke, or have you previously been smoking? |
| [11] "drugs" | Do you use, or have you previously been using drugs like hash, cocaine, heroin, amphetamine, morphine or LSD? |
| [12] "exercise___1" | Light exercise (walking, slow biking) |
| [13] "exercise___2" | moderate exercise (swimming, fast walking) |
| [14] "exercise___3" | Hard exercise (running, fast biking) |
| [15] "exercise___4" | Only daily exercise like house work or garden work |
| [16] "exercise___5" | I have a job that is physically hard |
| [17] "exercise___0" | no exercise |
| [18] "overuse" | Do you drink, or have you previously had an overuse of alcohol of more than 14 units a week for women or 21 units a week for men, for more than 5 years total? |
| [19] "alc_3m" | How many units of alcohol have you been drinking a week, for the last 3 months? |
| [20] "audit1" | How often do you drink alcohol? |
| [21] "audit2" | How many units do you normally have when drinking? |
| [22] "audit3" | How often do you have more than 5 units on the same occasion? |
| [23] "audit4" | Have you had trouble to stop drinking alcohol, after starting, within the last year? |
| [24] "audit5" | Have you had trouble to do what you have planned, because you have been drinking alcohol, within the last year? |
| [25] "audit6" | Have you needed alcohol in the morning, after drinking the night before, within the last year? |
| [26] "audit7" | Have you been feeling guilty of regrets after drinking alcohol, within the last year? |
| [27] "audit8" | Have you had trouble remembering what happened the night before, because you had been drinking alcohol, within the last year? |
| [28] "audit9" | Have you or somebody else been injured because you had been drinking alcohol? |
| [29] "audit10" | Have family members, friends, doctors or others been worried about your alcohol habits, or suggested you to lower your consumption? |
| [30] "drinkslastweek" | How many units of alcohol did you drink last week? |
| [31] "alc_wine" | Which type of alcohol do you prefer? Wine |
| [32] "alc_beer" | Which type of alcohol do you prefer? beer |
| [33] "alc_spirits" | Which type of alcohol do you prefer? spirits |
| [34] "alc_fortifiedwine" | Which type of alcohol do you prefer? fortified wine |
| [35] "hr" | heart rate in beats per minute |
| [36] "sbp" | systolic blood pressure |
| [37] "dbp" | diastolic blood pressure |
| [38] "map" | mean arterial pressure |
| [39] "weight" | weight in kilos |
| [40] "height" | height in centimeters |
| [41] "bmi" | body mass index in kg/m^2^ |
| [42] "bmi30" | bodymass index above 30 kg/m^2^ |
| [43] "muac" | Mid upper arm circumference in cm |
| [44] "waist" | waist circumference in cm |
| [45] "hip" | hip circumference in cm |
| [46] "whr" | hip-waist ratio |
| [47] "hgs1" | Hand-grip strength measurement 1 in lbs |
| [48] "hgs2" | Hand-grip strength measurement 2 in lbs |
| [49] "hgs3" | Hand-grip strength measurement 3 in lbs |
| [50] "hgs" | mean hand-grip strength in lbs |
| [51] "centralobesity" | abdominal obesity defined as BMI>30 or abdominal circumference >94 cm for men, or >80 cm for women |
| [52] "hypertension" | hypertension defined as >130 mmHg or >85mmHg, or medical treatment |
| [53] "dm_predm" | diabetes, or glucose intolerance defined as fasting glucose >5.6 or medical treatment |
| [54] "mets_points" | numner of metabolic risk factors either, centralobesity, high triglycerides, low hdl, hypertension or diabetes/ glucose intolerance |
| [55] "risk1___2" | diabetes as risk factor (based on dm_predm variable) |
| [56] "risk1___3" | metabolic syndrome as a risk factor (based on mets_points variable, counting 3 or more points) |
| [57] "risk1___4" | obesity as a risk factor (based on BMI>30) |
| [58] "hightrigly" | high triglycerides defined as triglycerides >1.7 mmol/L or medical treatment |
| [59] "lowhdl" | low HDL defined as 1.03 mmol/L for men, < 1.29 mmol/L for women or medical treatment |
| [60] "alt" | alanine transaminase (U/L) |
| [61] "alb" | albumin (g/L) |
| [62] "ast" | aspartate transaminase (U/L) |
| [63] "alk" | alkaline phosphatase (U/L) |
| [64] "bili" | bilirubin (mg/dL) |
| [65] "crp" | C-reactive protein (mg/L) |
| [66] "ferri" | ferritin (μg/L) |
| [67] "ggt" | gamma-glutamyl transferase (U/L) |
| [68] "glc" | glucose (mmol/L) |
| [69] "glcmean" | mean glucose (mmol/L) |
| [70] "hb" | hemoglobin (mmol/L) |
| [71] "hba1c" | hemoglobin A1c (mmol/mol) |
| [72] "hdl" | cholesterol hdl (mmol/mol) |
| [73] "iga" | Immunoglobulin A (g/l) |
| [74] "igg" | Immunoglobulin G (g/l) |
| [75] "igm" | Immunoglobulin M (g/L) |
| [76] "sod" | sodium (mmol/L) |
| [77] "inr" | international normalized ratio |
| [78] "chol" | cholesterol (mmol/L) |
| [79] "leu" | leucocytes (10E9/L) |
| [80] "mcv" | erythrocyte volume (fL) |
| [81] "meld" | model of end stage liver disease |
| [82] "trigly" | triglycerides (mmol/L) |
| [83] "trc" | platelets (10E9/L) |
| [84] "trfe" | Transferrin (μmol/L) |
| [85] "living" | What is your housing situation? |
| [86] "kids" | Do you have kids? |
| [87] "work" | what is your work situation? |
| [88] "education" | What is your latest graduated education? |
| [89] "income" | what is your income in DKK/year? |
| [90] "comorbid___0" | no comorbidity |
| [91] "comorbid___1" | COPD |
| [92] "comorbid___2" | other lung disease |
| [93] "comorbid___3" | hypercholesterolemia |
| [94] "comorbid___4" | hypertension |
| [95] "comorbid___8" | acute myocardial infarction |
| [96] "comorbid___9" | cardiac failure |
| [97] "comorbid___11" | cardiac bypass |
| [98] "comorbid___13" | stroke or TCI |
| [99] "comorbid___14" | diabetes type 1 |
| [100] "comorbid___15" | diabetes type 2 |
| [101] "comorbid___16" | hyper- or hypothyrodism |
| [102] "comorbid___19" | reflux |
| [103] "comorbid___20" | chronic pancreatitis |
| [104] "comorbid___22" | depression |
| [105] "comorbid___21" | irritable bowel disease |
| [106] "comorbid___23" | anxiety |
| [107] "comorbid___24" | chronic pain |
| [108] "comorbid___25" | previous malignant disease |
| [109] "comorbid___999" | other comorbidity |
| [110] "elev_enz" | Have you previously had raised liver function tests? |
| [111] "elev_enz_cons___0" | What actions were taken? no actions |
| [112] "elev_enz_cons___1" | What actions were taken? follow up at GP |
| [113] "elev_enz_cons___2" | What actions were taken? ultrasound scan through my GP |
| [114] "elev_enz_cons___3" | What actions were taken? hospital referral and examinations |
| [115] "elev_enz_concl___1" | What were the conculsion? no meaning |
| [116] "elev_enz_concl___2" | What were the conculsion? fatty liver |
| [117] "elev_enz_concl___3" | What were the conculsion? Signs of liver damage or cirrhosis |
| [118] "elev_enz_concl___4" | What were the conculsion? alcoholic hepatitis |
| [119] "elev_enz_concl___5" | What were the conculsion? I do not know |
| [120] "elev_enz_concl___999" | What were the conculsion? Other |
| [121] "fam_liver_disease" | Do anybody from your family have liver disease? |
| [122] "fam_liver_rel___1" | parents |
| [123] "fam_liver_rel___2" | siblings |
| [124] "fam_liver_rel___3" | kids |
| [125] "fam_liver_rel___4" | granparents |
| [126] "fam_liver_rel___5" | other relatives |
| [127] "fam_liver_rel___6" | partner |
| [128] "fam_alc" | Does anybody from your family have an alcohol overuse? |
| [129] "fam_alc_rel___1" | parents |
| [130] "fam_alc_rel___2" | siblings |
| [131] "fam_alc_rel___3" | kids |
| [132] "fam_alc_rel___4" | granparents |
| [133] "fam_alc_rel___5" | other relatives |
| [134] "fam_alc_rel___6" | partner |
| [135] "group" | recruitment group (alcohol, non-alcohol, background population) |
| [136] "dm_complications___29" | Albuminuri |
| [137] "dm_complications___30" | nefropathy |
| [138] "dm_complications___31" | retinopathy |
| [139] "dm_complications___32" | Neuropathy |
| [140] "dm_complications___33" | peripheral arterial disease |
| [141] "dm_complications___34" | macro angiopathy |
| [142] "dm_complications___0" | none |
| [143] "comorbid_cci___1" | Charlson comorbidity index: acute myocardial infarction |
| [144] "comorbid_cci___2" | Charlson comorbidity index: cardiac failure |
| [145] "comorbid_cci___3" | Charlson comorbidity index: arteriosclerosis |
| [146] "comorbid_cci___4" | Charlson comorbidity index: stroke or TCI |
| [147] "comorbid_cci___6" | Charlson comorbidity index: chronic lung disease |
| [148] "comorbid_cci___7" | Charlson comorbidity index: chronic connective tissue disease |
| [149] "comorbid_cci___8" | Charlson comorbidity index: gastric ulcer |
| [150] "comorbid_cci___9" | Charlson comorbidity index: mild liver disease |
| [151] "comorbid_cci___10" | Charlson comorbidity index: diabetes without complications |
| [152] "comorbid_cci___11" | Charlson comorbidity index: diabetes with organ complications |
| [153] "comorbid_cci___13" | Charlson comorbidity index: moderate-severe kidney disease |
| [154] "comorbid_cci___14" | Charlson comorbidity index: solid tumor (non metastatic) |
| [155] "comorbid_cci___15" | Charlson comorbidity index: Leukemia |
| [156] "comorbid_cci___16" | Charlson comorbidity index: Lymphoma, Multiple myeloma |
| [157] "comorbid_cci___17" | Charlson comorbidity index: Moderate – severe liver disease |
| [158] "comorbid_cci___18" | Charlson comorbidity index: Metastatic solid tumor |
| [159] "med_type___0" | no medication |
| [160] "med_type___1" | antihypertensive or diuretics |
| [161] "med_type___2" | blood thinner |
| [162] "med_type___3" | antidiabetic |
| [163] "med_type___4" | anticholesterol |
| [164] "med_type___5" | antidepressants or antianxiety |
| [165] "med_type___6" | pain reliever |
| [166] "med_type___7" | inhalation for COPD |
| [167] "med_type___8" | antacids |
| [168] "med_type___10" | laxative |
| [169] "med_type___11" | vitamins, minerals, nature medicine, supplements |
| [170] "med_type___12" | alcoholtreatment |
| [171] "med_type___999" | other medication |
| [172] "bp___1" | ACE blockers |
| [173] "bp___2" | Angiotensin receptor blockers |
| [174] "bp___3" | Calcium antagonist |
| [175] "bp___4" | Thiazid |
| [176] "bp___5" | Beta-blockers |
| [177] "bp___6" | other antihypertensiva |
| [178] "bp___7" | Furosemide |
| [179] "bp___8" | Spironolactone |
| [180] "bloodthinner___1" | acetylsalicylic acid |
| [181] "bloodthinner___2" | marevan |
| [182] "bloodthinner___3" | non-vitamin K oral anticoagulants |
| [183] "bloodthinner___4" | Platelet inhibitors |
| [184] "bloodthinner___999" | other blood thinning medication |
| [185] "med_dm___1" | Metformin |
| [186] "med_dm___2" | DDP-IV-inhibitors |
| [187] "med_dm___3" | Sulfonylureas |
| [188] "med_dm___5" | GLP-1-receptor agonists |
| [189] "med_dm___6" | SGLT-2 inhibitors |
| [190] "med_dm___8" | insulin |
| [191] "insulin___1" | short acting insulin |
| [192] "insulin___2" | long acting insulin |
| [193] "insulin___3" | mix insulin |
| [194] "antidep___1" | ssri |
| [195] "antidep___2" | TCA |
| [196] "antidep___3" | MAO inhibitors |
| [197] "antidep___4" | SNRI |
| [198] "antidep___6" | NaSSA |
| [199] "antidep___7" | benzodiazepine |
| [200] "antidep___8" | antipsychotics |
| [201] "antidep___999" | other antidepressants |
| [202] "pain___1" | Paracetamol |
| [203] "pain___2" | NSAID |
| [204] "pain___3" | tramadole, dolole |
| [205] "pain___4" | morphine |
| [206] "pain___5" | methadone |
| [207] "pain___999" | other pain relivers |
| [208] "nsaid___1" | Ibuprofene |
| [209] "nsaid___2" | Voltaren |
| [210] "nsaid___3" | acetylsalicylic acid |
| [211] "nsaid___4" | diclone |
| [212] "nsaid___5" | other NSAID |
| [213] "antacid___1" | Antacida |
| [214] "antacid___2" | H2-receptorantagonist |
| [215] "antacid___3" | protone pump inhibitors |
| [216] "antacid___4" | other antacida |
| [217] "affmiddel___1" | magnesia |
| [218] "affmiddel___2" | laxoberal |
| [219] "affmiddel___3" | Loperamid |
| [220] "affmiddel___4" | Laktulose |
| [221] "affmiddel___5" | Macrogol (Movicol) |
| [222] "affmiddel___6" | seed pods (HUSK) |
| [223] "affmiddel___999" | other laxatives |
| [224] "vitamins___1" | mulitvitamin |
| [225] "vitamins___2" | calcium and D-vitamine |
| [226] "vitamins___3" | magnesium |
| [227] "vitamins___4" | zink |
| [228] "vitamins___5" | b-combin vitamine |
| [229] "vitamins___6" | tiamin |
| [230] "vitamins___7" | fish oil |
| [231] "vitamins___8" | seed pods (HUSK) |
| [232] "vitamins___9" | c-vitamine |
| [233] "vitamins___999" | other vitamines |
| [234] "antibiotics" | Have you had antibiotics within the last 6 months? |

**2. Structure of the ensemble learning algorithms**


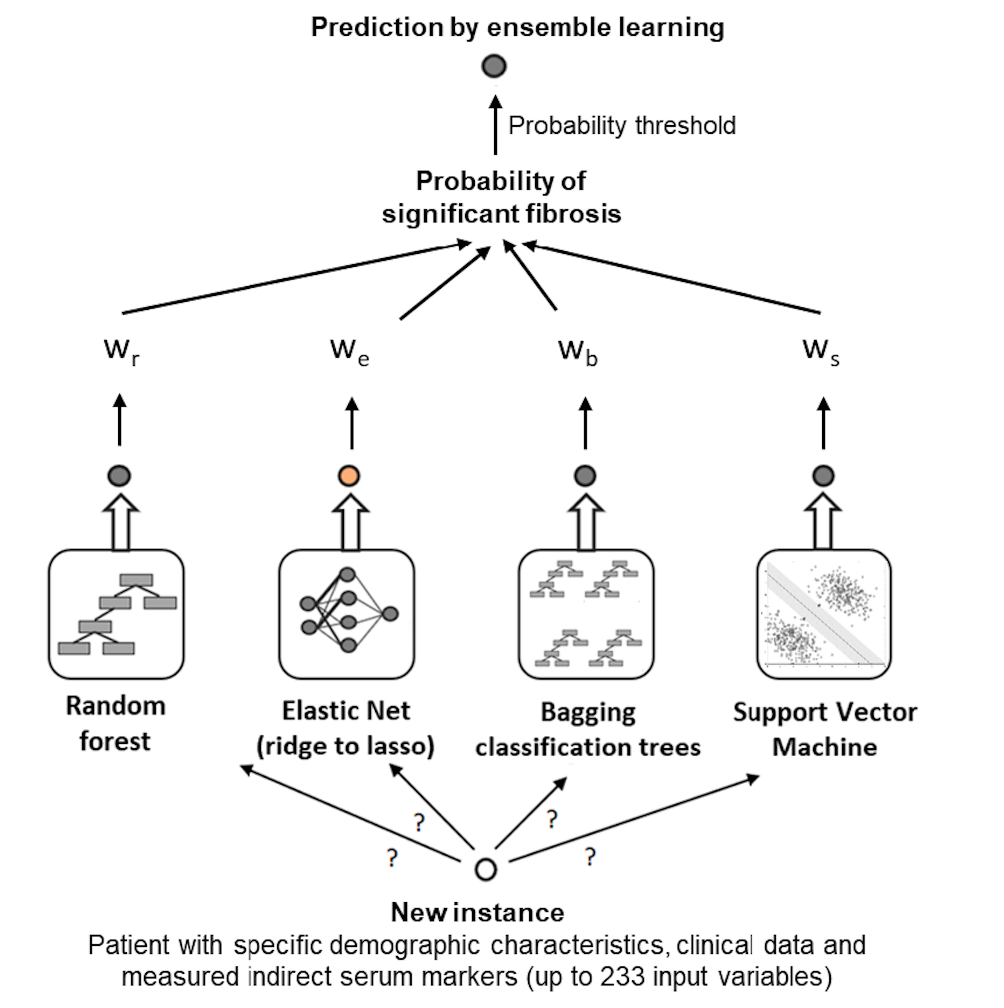


**Structure of the ensemble learning algorithms**, including four base learners (Random forest, Elastic Net, Bagging classification tress and Support Vector Machine) and their corresponding optimized weights (w_r_, w_e_, w_b_, and w_s_).

The hyperparameters of the base learners are: random forest (mtry=number of variables randomly sampled as candidates at each split, ntree=number of trees to grow), elastic Net (alpha = elastic net mixing parameter, range [0, 1]. 0 = ridge regression and 1 = lasso), bagging classification tress (nbagg = number of bootstrap replications) and support vector machine (C=regularization term and gamma=parameter in the radial basis function kernel)

**3. Further description of data splits and pre-processing**

With a view to reliably assessing the generalization error, we performed a series of data splits. First, we randomly extracted a subset of the data (hold-out dataset), which included 10% of the available dataset. The holdout dataset was not used in any way for training, validation or testing, and this unseen data was exclusively reserved to perform the final assessment of the model’s predictive performance. In order to build a substantially predictable and robust model, we applied a repeated random subsampling (RRS) strategy in which the remaining dataset was repeatedly and randomly split (5 times, resulting in 5 repetitions) into training (60%), validation (20%) and testing (20%). As in many other medical real-world studies, our dataset was imbalanced (ratio=7:1). In order to maximize correct identification in the target (minority) class, synthetic samples were generated, only in the training dataset, using SMOTE (Synthetic Minority Over-sampling Technique). (Chawla et al., 2002)

In each LiverAID model (XXS to 4XL), the optimal values for the hyperparameters for each sampling repetition (1 to 5) were obtained using a procedure that iteratively assessed the cross-validated performance of the algorithms in the training set over a range of plausible hyperparameter values (Wong et al., 2019). The final hyperparameters for each of LiverAID model were selected using majority voting across the 5 repetitions of the training dataset. The models using these final hyperparameters values were then trained on the training datasets of each repetition, to obtain the final models. The validation sets were then used to optimize the probability threshold value that resulted in an NPV≥98%, while the testing datasets were used to get a preliminary estimation of the generalization error. Finally, the predictive performance of each of the LiverAID models was evaluated on the completely unseen hold-out dataset.

Chawla N, Bowyer K, Hall L, Kegelmeyer W. SMOTE: Synthetic Minority Over-sampling Technique. J Artif Intell Res (JAIR). 2002;16:321-57.

Wong J, Manderson T, Abrahamowicz M, Buckeridge DL, Tamblyn R. Can Hyperparameter Tuning Improve the Performance of a Super Learner?: A Case Study. Epidemiology (Cambridge, Mass). 2019;30(4):521-31.

**4. Liver stiffness status estimated from transient elastography measurements of the subjects included in the study**

| Number of subjects whose liver stiffness measurement (LSM) is >8 kPa (corresponding to “significant liver stiffness”) and number of subjects whose LSM is ≤8 kPa (corresponding to “not significant liver stiffness”) in each data subset and repetition. | | | | | | | | | | |
| --- | --- | --- | --- | --- | --- | --- | --- | --- | --- | --- |
| Data split | Training | | Training after SMOTE | | Validation | | Testing | | Hold-out | |
|  | LSM≤8 kPa | LSM>8 kPa | LSM≤8 kPa | LSM>8 kPa | LSM≤8 kPa | LSM>8 kPa | LSM≤8 kPa | LSM>8 kPa | LSM≤8 kPa | LSM>8 kPa |
| Repetition 1 | 1607 | 203 | 406 | 406 | 536 | 67 | 519 | 85 | 287 | 48 |
| Repetition 2 | 1591 | 219 | 438 | 438 | 538 | 65 | 533 | 71 |  |  |
| Repetition 3 | 1604 | 206 | 412 | 412 | 535 | 68 | 523 | 81 |  |  |
| Repetition 4 | 1590 | 220 | 440 | 440 | 537 | 66 | 535 | 69 |  |  |
| Repetition 5 | 1601 | 209 | 418 | 418 | 532 | 71 | 529 | 75 |  |  |

**5. Area Under the Curve (AUC) of the Receiver Operating Characteristic curve (ROC) of LiverAID models predicting significant liver stiffness (LSM>8kPa) in validation, testing and hold-out datasets**

| **Area Under the Curve (AUC) of the Receiver Operating Characteristic curve (ROC) of LiverAID models predicting significant liver stiffness (LSM>8kPa) in validation, testing and hold-out datasets** | | | | | | | | | | | | | | |
| --- | --- | --- | --- | --- | --- | --- | --- | --- | --- | --- | --- | --- | --- | --- |
|  | **Repetition** | **LiverAID XXS** | | **LiverAID XS** | | **LiverAID S** | | **LiverAID M** | | **LiverAID L** | | **LiverAID 4XL** | |  |
| **Validation** | 1 | 0.84 | (0.78-0.89) | 0.88 | (0.82-0.91) | 0.88 | (0.84-0.93) | 0.89 | (0.84-0.93) | 0.89 | (0.85-0.94) | 0.91 | (0.86-0.95) |  |
|  | 2 | 0.83 | (0.77-0.89) | 0.90 | (0.86-0.93) | 0.90 | (0.88-0.95) | 0.91 | (0.87-0.94) | 0.91 | (0.87-0.95) | 0.93 | (0.89-0.97) |  |
|  | 3 | 0.87 | (0.82-0.92) | 0.88 | (0.84-0.92) | 0.87 | (0.85-0.94) | 0.90 | (0.86-0.94) | 0.91 | (0.88-0.95) | 0.93 | (0.90-0.96) |  |
|  | 4 | 0.87 | (0.82-0.92) | 0.88 | (0.83-0.93) | 0.91 | (0.88-0.95) | 0.92 | (0.88-0.96) | 0.93 | (0.89-0.97) | 0.93 | (0.90-0.97) |  |
|  | 5 | 0.88 | (0.84-0.92) | 0.89 | (0.85-0.93) | 0.91 | (0.87-0.95) | 0.92 | (0.88-0.95) | 0.91 | (0.87-0.95) | 0.92 | (0.88-0.96) |  |
|  | Mean±SD | 0.86±0.02 | | 0.88±0.01 | | 0.89±0.02 | | 0.91±0.01 | | 0.91±0.01 | | 0.93±0.01 | |  |
| **Testing** | 1 | 0.89 | (0.85-0.92) | 0.89 | (0.85-0.92) | 0.89 | (0.86-0.93) | 0.90 | (0.87-0.93) | 0.89 | (0.85-0.92) | 0.93 | (0.90-0.96) |  |
|  | 2 | 0.87 | (0.82-0.91) | 0.87 | (0.82-0.91) | 0.90 | (0.86-0.94) | 0.91 | (0.88-0.95) | 0.91 | (0.87-0.95) | 0.94 | (0.91-0.97) |  |
|  | 3 | 0.85 | (0.80-0.90) | 0.88 | (0.83-0.92) | 0.90 | (0.86-0.94) | 0.91 | (0.87-0.95) | 0.91 | (0.87-0.95) | 0.92 | (0.88-0.96) |  |
|  | 4 | 0.87 | (0.82-0.92) | 0.87 | (0.82-0.92) | 0.87 | (0.84-0.93) | 0.89 | (0.84-0.94) | 0.90 | (0.85-0.94) | 0.91 | (0.87-0.96) |  |
|  | 5 | 0.87 | (0.82-0.92) | 0.91 | (0.87-0.95) | 0.93 | (0.90-0.97) | 0.93 | (0.90-0.96) | 0.92 | (0.88-0.96) | 0.94 | (0.91-0.98) |  |
|  | Mean±SD | 0.87±0.01 | | 0.88±0.02 | | 0.90±0.02 | | 0.91±0.01 | | 0.90±0.01 | | 0.93±0.01 | |  |
| **Hold-out** | 1 | 0.85 | (0.80-0.92) | 0.87 | (0.81-0.93) | 0.91 | (0.88-0.97) | 0.92 | (0.88-0.96) | 0.93 | (0.88-0.97) | 0.94 | (0.88-0.98) |  |
|  | 2 | 0.86 | (0.80-0.92) | 0.89 | (0.84-0.94) | 0.91 | (0.87-0.97) | 0.92 | (0.87-0.96) | 0.93 | (0.88-0.97) | 0.94 | (0.89-0.99) |  |
|  | 3 | 0.85 | (0.80-0.92) | 0.90 | (0.85-0.94) | 0.90 | (0.87-0.96) | 0.91 | (0.86-0.96) | 0.92 | (0.88-0.97) | 0.93 | (0.89-0.98) |  |
|  | 4 | 0.86 | (0.80-0.92) | 0.88 | (0.82-0.94) | 0.90 | (0.87-0.96) | 0.91 | (0.86-0.96) | 0.91 | (0.85-0.96) | 0.93 | (0.88-0.98) |  |
|  | 5 | 0.86 | (0.80-0.91) | 0.89 | (0.84-0.94) | 0.91 | (0.86-0.96) | 0.92 | (0.87-0.97) | 0.92 | (0.87-0.96) | 0.94 | (0.89-0.99) |  |
|  | Mean±SD | 0.86±0.01 | | 0.89±0.01 | | 0.91±0.01 | | 0.92±0.00 | | 0.92±0.01 | | 0.94±0.00 | |  |
| 95% CI of the estimated AUC are shown in parenthesis.  Mean±SD indicate the mean and standard deviation of the AUC across repetitions (resulting from using different data splits into training, validation and testing following the repeated random subsampling strategy)  The predictive performance of our LiverAID models in the validation, testing and hold-out dataset, indicate relatively good ability to generalize. | | | | | | | | | | | | | | |

**6. P-values for the comparison of AUC between LiverAID models and standard blood-based indices in predicting significant liver stiffness (LSM>8kPa)**

| P-values for the AUC comparison between all models (Repetition 1) | | | | | | | | | | |
| --- | --- | --- | --- | --- | --- | --- | --- | --- | --- | --- |
|  | FIB-4 | Forns | APRI | FIB-4 + Forns + APRI | LiverAID  XXS | LiverAID  XS | LiverAID  S | LiverAID  M | LiverAID  L | LiverAID 4XL |
| FIB-4 | - |  |  |  |  |  |  |  |  |  |
| Forns | 0.987 | - |  |  |  |  |  |  |  |  |
| APRI | 0.867 | 0.998 | - |  |  |  |  |  |  |  |
| FIB-4 + Forns + APRI | **0.021** | **0.003** | 0.173 | - |  |  |  |  |  |  |
| LiverAID XXS | **0.000** | **0.000** | **0.001** | **0.008** | - |  |  |  |  |  |
| LiverAID XS | **0.000** | **0.000** | **0.001** | **0.008** | 0.430 | - |  |  |  |  |
| LiverAID S | **0.000** | **0.000** | **0.000** | **0.000** | **0.005** | **0.001** | - |  |  |  |
| LiverAID M | **0.000** | **0.000** | **0.000** | **0.000** | **0.001** | **0.003** | 0.351 | - |  |  |
| LiverAID L | **0.000** | **0.000** | **0.000** | **0.000** | **0.004** | **0.001** | 0.482 | 0.303 | - |  |
| LiverAID 4XL | **0.000** | **0.000** | **0.000** | **0.000** | **0.003** | **0.001** | 0.219 | 0.126 | 0.152 | - |

| P-values for the AUC comparison between all models (Repetition 2) | | | | | | | | | | |
| --- | --- | --- | --- | --- | --- | --- | --- | --- | --- | --- |
|  | FIB-4 | Forns | APRI | FIB-4 + Forns + APRI | LiverAID  XXS | LiverAID  XS | LiverAID  S | LiverAID  M | LiverAID  L | LiverAID 4XL |
| FIB-4 | - |  |  |  |  |  |  |  |  |  |
| Forns | 0.987 | - |  |  |  |  |  |  |  |  |
| APRI | 0.867 | 0.998 | - |  |  |  |  |  |  |  |
| FIB-4 + Forns + APRI | **0.021** | **0.003** | 0.173 | - |  |  |  |  |  |  |
| LiverAID XXS | **0.000** | **0.000** | **0.000** | **0.002** | - |  |  |  |  |  |
| LiverAID XS | **0.000** | **0.000** | **0.000** | **0.002** | 0.211 | - |  |  |  |  |
| LiverAID S | **0.000** | **0.000** | **0.000** | **0.000** | **0.017** | **0.014** | - |  |  |  |
| LiverAID M | **0.000** | **0.000** | **0.000** | **0.000** | **0.009** | *0.072* | 0.365 | - |  |  |
| LiverAID L | **0.000** | **0.000** | **0.000** | **0.000** | **0.004** | **0.007** | 0.256 | 0.172 | - |  |
| LiverAID 4XL | **0.000** | **0.000** | **0.000** | **0.000** | **0.004** | **0.009** | *0.059* | **0.049** | 0.157 | - |

| P-values for the AUC comparison between all models (Repetition 3) | | | | | | | | | | |
| --- | --- | --- | --- | --- | --- | --- | --- | --- | --- | --- |
|  | FIB-4 | Forns | APRI | FIB-4 + Forns + APRI | LiverAID  XXS | LiverAID  XS | LiverAID  S | LiverAID  M | LiverAID  L | LiverAID 4XL |
| FIB-4 | - |  |  |  |  |  |  |  |  |  |
| Forns | 0.987 | - |  |  |  |  |  |  |  |  |
| APRI | 0.867 | 0.998 | - |  |  |  |  |  |  |  |
| FIB-4 + Forns + APRI | **0.021** | **0.003** | 0.173 | - |  |  |  |  |  |  |
| LiverAID XXS | **0.000** | **0.000** | **0.001** | **0.006** | - |  |  |  |  |  |
| LiverAID XS | **0.000** | **0.000** | **0.000** | **0.001** | *0.086* | - |  |  |  |  |
| LiverAID S | **0.000** | **0.000** | **0.000** | **0.000** | **0.028** | 0.102 | - |  |  |  |
| LiverAID M | **0.000** | **0.000** | **0.000** | **0.000** | **0.010** | 0.132 | 0.423 | - |  |  |
| LiverAID L | **0.000** | **0.000** | **0.000** | **0.000** | **0.010** | *0.058* | 0.187 | 0.240 | - |  |
| LiverAID 4XL | **0.000** | **0.000** | **0.000** | **0.000** | **0.005** | **0.041** | *0.079* | *0.088* | 0.141 | - |

| P-values for the AUC comparison between all models (Repetition 4) | | | | | | | | | | |
| --- | --- | --- | --- | --- | --- | --- | --- | --- | --- | --- |
|  | FIB-4 | Forns | APRI | FIB-4 + Forns + APRI | LiverAID  XXS | LiverAID  XS | LiverAID  S | LiverAID  M | LiverAID  L | LiverAID 4XL |
| FIB-4 | - |  |  |  |  |  |  |  |  |  |
| Forns | 0.987 | - |  |  |  |  |  |  |  |  |
| APRI | 0.867 | 0.998 | - |  |  |  |  |  |  |  |
| FIB-4 + Forns + APRI | **0.021** | **0.003** | 0.173 | - |  |  |  |  |  |  |
| LiverAID XXS | **0.000** | **0.000** | **0.001** | **0.004** | - |  |  |  |  |  |
| LiverAID XS | **0.000** | **0.000** | **0.001** | **0.008** | 0.345 | - |  |  |  |  |
| LiverAID S | **0.000** | **0.000** | **0.000** | **0.001** | **0.026** | **0.016** | - |  |  |  |
| LiverAID M | **0.000** | **0.000** | **0.000** | **0.000** | **0.016** | **0.039** | 0.485 | - |  |  |
| LiverAID L | **0.000** | **0.000** | **0.000** | **0.000** | *0.061* | *0.067* | 0.292 | 0.291 | - |  |
| LiverAID 4XL | **0.000** | **0.000** | **0.000** | **0.000** | **0.006** | **0.041** | **0.044** | *0.078* | **0.014** | - |

| P-values for the AUC comparison between all models (Repetition 5) | | | | | | | | | | |
| --- | --- | --- | --- | --- | --- | --- | --- | --- | --- | --- |
|  | FIB-4 | Forns | APRI | FIB-4 + Forns + APRI | LiverAID  XXS | LiverAID  XS | LiverAID  S | LiverAID  M | LiverAID  L | LiverAID 4XL |
| FIB-4 | - |  |  |  |  |  |  |  |  |  |
| Forns | 0.987 | - |  |  |  |  |  |  |  |  |
| APRI | 0.867 | 0.998 | - |  |  |  |  |  |  |  |
| FIB-4 + Forns + APRI | **0.021** | **0.003** | 0.173 | - |  |  |  |  |  |  |
| LiverAID XXS | **0.000** | **0.000** | **0.001** | **0.003** | - |  |  |  |  |  |
| LiverAID XS | **0.000** | **0.000** | **0.000** | **0.001** | *0.065* | - |  |  |  |  |
| LiverAID S | **0.000** | **0.000** | **0.000** | **0.001** | **0.031** | *0.080* | - |  |  |  |
| LiverAID M | **0.000** | **0.000** | **0.000** | **0.000** | **0.003** | **0.027** | 0.300 | - |  |  |
| LiverAID L | **0.000** | **0.000** | **0.000** | **0.000** | **0.020** | *0.058* | 0.337 | 0.440 | - |  |
| LiverAID 4XL | **0.000** | **0.000** | **0.000** | **0.000** | **0.002** | **0.003** | *0.067* | *0.063* | **0.039** | - |

p-values < 0.05 are indicated in bold.

p-values = 0.05-0.10 are shown in cursive.
